# Multimodal deep learning methods enhance genomic prediction of wheat breeding

**DOI:** 10.1093/g3journal/jkad045

**Published:** 2023-02-27

**Authors:** Abelardo Montesinos-López, Carolina Rivera, Francisco Pinto, Francisco Piñera, David Gonzalez, Mathew Reynolds, Paulino Pérez-Rodríguez, Huihui Li, Osval A Montesinos-López, Jose Crossa

**Affiliations:** Departamento de Matemáticas, Centro Universitario de Ciencias Exactas e Ingenierías (CUCEI), Universidad de Guadalajara, 44430, Guadalajara, Jalisco, Mexico; International Maize and Wheat Improvement Center (CIMMYT), Carretera México- Veracruz Km. 45, El Batán, CP 56237, Texcoco, Edo. de México, Mexico; International Maize and Wheat Improvement Center (CIMMYT), Carretera México- Veracruz Km. 45, El Batán, CP 56237, Texcoco, Edo. de México, Mexico; International Maize and Wheat Improvement Center (CIMMYT), Carretera México- Veracruz Km. 45, El Batán, CP 56237, Texcoco, Edo. de México, Mexico; International Maize and Wheat Improvement Center (CIMMYT), Carretera México- Veracruz Km. 45, El Batán, CP 56237, Texcoco, Edo. de México, Mexico; International Maize and Wheat Improvement Center (CIMMYT), Carretera México- Veracruz Km. 45, El Batán, CP 56237, Texcoco, Edo. de México, Mexico; Colegio de Postgraduados, Montecillos, Edo. de México, CP 56230, Mexico; Institute of Crop Sciences, The National Key Facility for Crop Gene Resources and Genetic Improvement and CIMMYT China office, Chinese Academy of Agricultural Sciences, Beijing, 100081, China; Facultad de Telemática, Universidad de Colima, C. P. 28040, Colima, Estado de Colima, México; International Maize and Wheat Improvement Center (CIMMYT), Carretera México- Veracruz Km. 45, El Batán, CP 56237, Texcoco, Edo. de México, Mexico; Colegio de Postgraduados, Montecillos, Edo. de México, CP 56230, Mexico

**Keywords:** conventional genomic prediction method, novel deep learning method, genomic prediction accuracy (GP accuracy)

## Abstract

While several statistical machine learning methods have been developed and studied for assessing the genomic prediction (GP) accuracy of unobserved phenotypes in plant breeding research, few methods have linked genomics and phenomics (imaging). Deep learning (DL) neural networks have been developed to increase the GP accuracy of unobserved phenotypes while simultaneously accounting for the complexity of genotype–environment interaction (GE); however, unlike conventional GP models, DL has not been investigated for when genomics is linked with phenomics. In this study we used 2 wheat data sets (DS1 and DS2) to compare a novel DL method with conventional GP models. Models fitted for DS1 were GBLUP, gradient boosting machine (GBM), support vector regression (SVR) and the DL method. Results indicated that for 1 year, DL provided better GP accuracy than results obtained by the other models. However, GP accuracy obtained for other years indicated that the GBLUP model was slightly superior to the DL. DS2 is comprised only of genomic data from wheat lines tested for 3 years, 2 environments (drought and irrigated) and 2–4 traits. DS2 results showed that when predicting the irrigated environment with the drought environment, DL had higher accuracy than the GBLUP model in all analyzed traits and years. When predicting drought environment with information on the irrigated environment, the DL model and GBLUP model had similar accuracy. The DL method used in this study is novel and presents a strong degree of generalization as several modules can potentially be incorporated and concatenated to produce an output for a multi-input data structure.

## Introduction

The principle of genomic selection (GS) and genomic prediction (GP) is to build an accurate prediction model based on a training population comprised of individuals with both genotypic and phenotypic data to predict unobserved lines. Extensive research studies were conducted and novel statistical methods that incorporate pedigree, genomic, and environmental covariates (e.g. weather data) into statistical–genetic prediction models have been developed, studied, and employed (Crossa *et al*. 2021). Genomic Best Linear Unbiased Predictor (GBLUP) models are widely used in GP, and the extension of GBLUP for incorporating genotype × environment (GE) interaction improved the accuracy of predicting unobserved cultivars in environments. Jarquín *et al*. (2014) found that the prediction accuracy of models, including the GE term (reaction norm model), was substantially higher (17–34%) than that of models based only on major effects. For a maize ordinal data set, A. Montesinos-López *et al*. (2017) reported that models including GE achieved gains of 9–14% in prediction accuracy over models including only main effects. Using wheat data, Cuevas *et al*. (2016) found that models with the GE term were up to 60–68% better in terms of GP accuracy (association between the true genotypic values and the genomic genotypic values) than the corresponding single-environment models.

The genomic model that includes GE has been successfully used in several wheat and cotton applications, as exemplified by Pérez-Rodríguez and de los Campos (2014), who used only pedigree relationship information and environmental covariables, and Crossa *et al*. (2016), who used only markers in large sets of wheat gene bank landraces. Velu *et al*. (2016) applied the reaction norm model in wheat lines from CIMMYT's biofortification breeding program, with Zn and Fe content in the grain and other agronomic traits measured during two successive crop seasons (2011–2012 and 2012–2013). The prediction accuracy of Zn ranged between 0.331 and 0.694, with an average of 0.542. Prediction ability for Zn was more effective in high Zn environments when compared to low Zn environments. Prediction accuracy for Fe ranged from 0.324 to 0.734 with a mean prediction of 0.529 across environments.

Continued increases in GP accuracies reflect the success of genomic data for establishing genomic-assisted plant breeding (Bonnett *et al*. 2022; Beyene *et al*. 2015) and, subsequently, for increasing crop productivity in less time. Recently, studies have shown the benefits of integrating modern technologies into breeding programs (Crossa *et al.* 2021; A. Montesinos-López *et al*. 2017, 2018; O.A. Montesinos-López *et al*. 2019; Costa-Neto *et al.* 2021a, 2021b). This includes both genomics and detailed trait analysis using advances in phenomics (e.g. image or high-throughput phenotype, HTP) and environment characterization to increase the genomic prediction, thus accelerating future increases in genetic gains and crop production. There is empirical evidence showing that integrating HTP information with GS data has the potential to complement GS and increase crop productivity. One of the advantages of recent phenotyping technology is that it can quickly and accurately obtain data on many agronomic traits.

The primary goal of plant imaging HTP is to measure the different physiological stages of the plant through automated processes using digital camera technology that collects reflectance data at different wavelengths. This data can predict physiological or agronomic traits in plants with scores of spectral vegetative indices that are used as predictors. The reflectance data can also be used directly to predict the traits of interest. Using images to improve GP accuracy was highlighted by A. Montesinos-López *et al*. (2017), who proposed Bayesian functional regression models considering all available image bands, genomic or pedigree information, the main effects of lines and environments, as well as GE and the band × environment (BE) interaction effects. The authors observed that the models with BE interaction terms were the most accurate, while the functional regression models and the conventional models performed similarly in terms of prediction accuracy. Functional regression models are more parsimonious and computationally very efficient.

The interdisciplinary researchers in computing statistics and data science focus on achieving more accurate GP by using various statistical models. In this regard, deep neural network methods are powerful prediction tools in machine learning. Recent developments in deep neural networks and faster computer processing have made it possible to add new layers to the neural network (deep machine learning, DL) to capture small cryptic correlations between inputs, which in GP are interactions between markers that reflects the gene × gene and higher-order interaction (genetic epistasis). Initial applications of machine learning and neural networks in animal and plants GP were demonstrated by Gianola *et al*. (2011), González-Camacho *et al*. (2012), González-Camacho *et al*. (2016) and González-Recio and Forni (2011), O.A. Montesinos-López *et al*. (2018), and O.A. Montesinos-López *et al.* (2019).

Recently, several applications of GP using DL have been studied and compared with other methods (O.A. Montesinos-López *et al.* 2019). O.A. Montesinos-López *et al*. (2018) used the prediction performance of DL applied to multi-trait model and compared it to the performance of the Bayesian multi-trait and multi-environment (BMTME) model. The authors found that when the GE term was excluded, the best predictions were observed under the DL multi-trait model; however, when the GE term was considered, the BMTME outperformed the DL multi-trait. Using 9 data sets, O.A. Montesinos-López *et al*. (2018) found that when the GE interaction term was excluded, the DL method was better than the GBLUP model in 6 out of the 9 data sets. However, with the inclusion of GE, the GBLUP model was the best in 8 out of 9 data sets. O.A. Montesinos-López *et al*. (2018) compared GBLUP, univariate and multi-trait DL and found that when the GE was included, the best predictions were observed under the GBLUP model, while the worst were under the univariate DL model. However, when the GE was ignored, the best predictions were observed under the GBLUP method and the multi-trait DL model.

The most applied DL architectures in GS are the multilayer perceptron and the convolutional neural networks (CNN) (Jiang and Li 2020; O.A. Montesinos-López *et al.* 2021a, 2021b). Although thousands of single nucleotide polymorphisms (SNP) can be used in both DL methods, to avoid training a model with a vast number of parameters, the squared root matrix or the Cholesky factor of the genomic relations matrix is used. Another less computationally intensive option is the recently proposed method of Nazzicari and Biscarini (2022), which uses genomic data as an image for input data in the CNN by first ordering the data through hierarchical clustering. This last option could be used under a multimodal strategy where for each type of information, an individual neural network is created and its outputs are used as inputs in another output layer to produce the prediction value (Ouyang *et al*. 2014; Baltrušaitis *et al*. 2018). For an early overview on deep multimodal learning models, see Ramachandram and Taylor (2017), who explore its applications in diverse research fields. For example, in agriculture, Danilevicz *et al*. (2021) employed multimodal DL to integrate management and genotype information with the multispectral data to describe plant conditions during the field trial. A recent study integrated genomic, environment, and management data in a multimodal DL and found that multimodal DL and GBLUP, which included several interactions, had the best overall performance (Kick *et al*. 2023). Likewise, in the field of healthcare, multimodal learning models have been widely used (Huang *et al*. 2020; Venugopalan *et al*. 2021; Stahlschmidt *et al.* 2022; Kline *et al*. 2022).

Based on the previous considerations, the main objectives of this study were to compare the GP accuracy of several statistical machine learning models: (1) the conventional Bayesian GBLUP model, implemented by means of the Bayesian generalized linear regression (BGLR) R package (Pérez-Rodríguez and de los Campos 2014), (2) a novel multimodal genomic DL (MMDL) method that uses the creation of an individual neural network (as opposed to combining all available features types to feed a network) that combines the outputs to create the output value, (3) the gradient boosting machine (GBM) that can be used with regression (Hastie *et al*. 2009), and (4) the support vector regression (SVR) as a scheme to predict continuous values (Drucker *et al*. 1996).

Two wheat data sets were used in this study. Data set 1 (DS1) includes 350 wheat lines and incorporates genomic and image (NDVI) values as covariables that were measured several times at the vegetative (VG, 4 times) and grain filing (GF, 2 times) periods; both measurements were used on the same date of each year (aligned), using the average of these covariables on both periods. Furthermore, DS1 was analyzed over 2 years (cycles 2015–2016 and 2016–2017) using 2 traits: grain yield (YLD, grams per square meter) and thousand grains weight (TGW). Genomic prediction of DS1 were performed with GBLUP, MMDL, GBM, and SVR. DS2 was analyzed over 3 years; between 145 and 155 lines were tested in 2 environments (drought and irrigated) and 2 or 4 traits were measured. DS2 includes genomics but does not include image data. We predicted 1 entire year with the other using GBLUPs and deep learning (DL) methods with genomic information as the unique predictor.

## Materials and methods

This section has 5 subsections, each one describing (1) the structure of the phenotypic data sets, (2) how the metrics for assessing the prediction accuracy were computed, (3) the genotypic data, (4) the aerial high-throughput phenotyping used for DS1, and (5) the statistical models. The Appendix details how the two phenotypic data sets, DS1 and DS2, were adjusted for the experimental design in each year and environment and for each trait.

### Phenotypic data

#### Data set 1 (DS1)

The data set is a panel of 350 wheat cultivars and wheat accessions from a wheat gene bank, evaluated by the Physiology Unit of the Global Wheat Program at the International Center for Maize and Wheat Improvement (CIMMYT) over 2 years, using cycles 2015–2016 and 2016–2017 for 2 traits—grain yield (YLD, grams per square meter) and thousand grains weight (TGW)—under yield potential conditions (fully irrigated, timely sown). As already mentioned, data set 1 used genomic and image (NDVI) values as covariables; these were measured several times at the vegetative and grain filing periods and both measurements were used for (1) the same date of each year (aligned) or (2) the average of these covariables on both measurements’ periods. An alpha lattice design was used with 2 replicates and 6 incomplete blocks of size 5. The bands were measured 4 times during the vegetative (VG) period and 2 times during the grain filling (GF).

#### Data set 2 (DS2)

This data set is a panel of synthetic hexaploidy wheat lines that were formed by the hybridization and doubling of the cross between durum wheat and *Aegilops tauschii*, which were evaluated over 3 consecutive years (2015–2016, 2016–2017, and 2017–2018) by the Physiology Unit of the Global Wheat Program at CIMMYT. For each year, between 145 and 155 lines were tested in 2 kinds of environments, drought (suboptimal yield potential conditions) and irrigated (optimal yield potential conditions), and 2 or 4 traits were measured (biomass = BM, harvest index = HI, a thousand kernel weight = PMG, and grain yield = YLD). To predict each trait in one environment with information from the other environment for each year, methods GBLUPs and DL were used with *G* as the unique predictor.

### Assessing prediction accuracy

The prediction accuracy of several models was assessed by two methods: (1) predicting the performance of 1 entire year using all the lines from another year (leave-one-year out, LOO) and (2) a 5-fold cross-validation (5FCV) considering a training set of 80% of the wheat lines that predict 20% of the testing set comprised of unobserved wheat lines. Note that the multimodal DL model is only used under the 5FCV where the genomic and year predictors were included.

For DS2, no information on images were available for this data set, and for each year the prediction accuracy was assessed by predicting the performance of 1 entire environment (irrigated or drought) using all the lines from the other environment (LOO). Consequently, for this data set the multimodal DL resumes to the standard DL as module year (environment) and its interaction with the year is not applied when using the LOO cross-validation method.

### Genotyping data

For both panels, the DNA extraction was performed using the commercial kit and were genotyped using the 35 K Axiom Wheat Breeders array (Affymetrix, High Wycombe, UK) (Allen *et al*. 2017). For each marker, the genotype for each line was coded as the number of copies of a designated marker-specific allele carried by the line (absence = 0 and presence = 1). SNP markers with unexpected heterozygous >10% were removed; remaining heterozygous genotypes were recoded as either AA or BB according to the allele with higher frequency. Those markers that had more than 20% missing values or with minor allele frequency (MAF) < 0.05 were removed. Residual missing genotypes were imputed using mean imputation. A total of 10,560 and 10,064 SNPs were still available for analysis after quality control and imputation for DS1 and DS2, respectively.

### Aerial high-throughput phenotyping for DS1

Aerial multispectral images were collected using UAVs over the trials. In year 2015–2016, multispectral images were collected at 65 m above the ground using a MultiSpec 4C camera (SenseFly/Airinov, France) mounted on an eBee fixed-wings UAV (SenseFly Ltd., Switzerland). During years 2015–2016, the aerial imagery was collected at 60 m above ground level using the AscTec Falcon 8 multirotor UAV (Ascending Technologies, Germany) equipped with an ADC Lite multispectral camera (Tetracam, USA).

The multiple images were collected over the trials flying the drones in north–south flight lines with a lateral and longitudinal overlap of ∼80% of the field of view. Data collection took place under clear skies around noon. The images were used to reconstruct mosaics of the trials using the Pix4D Mapper software (Pix4D, Switzerland). The geo-referencing of each mosaic was achieved using ground control points distributed in the field. The spectral reflectance was calculated using calibration panels and then the NDVI was calculated using the RED and NIR bands. The NDVI orthomosaics were exported to ArcGIS (ESRI, USA) where a grid of polygons representing each plot was adjusted on top of the image. Before data extraction, the polygons were buffered 30 cm from each side to avoid the border effect and soil pixels. Using the “raster” package in R, the value of NDVI per plot was calculated by averaging all the pixels enclosed by each polygon and discarding possible outliers and soil pixels.

### Statistical and machine learning models with cross-validation

#### The GBLUP model with 5FCV and LOO cross-validation

The statistical GBLUP model used in this study assumed that each response variable is modeled as


(1) $$Y_{ij}=\mu +\mathrm{yea}r_{i}+g_{i}+\mathrm{year}\times g_{i}+\overset{4}{\underset{l=1}{\sum }}\;\beta_{\mathrm{vg},l}x_{\mathrm{vg},jl}+\overset{2}{\underset{l=1}{\sum }}\;\beta_{\mathrm{gf},l}x_{\mathrm{gf},jl}+\epsilon_{ij}$$

were $Y_{ij}$ is the value of the response variable of line jth measured in year ith, μ is the general mean, $\mathrm{yea}r_{i}$ is the fixed effect of the year ith(i=1,…,I), $g_{j}(j=1,\ldots ,J$) is the random effects of lines, $\mathrm{year}\times g_{ij}$ is the random interaction year × line effect, $\beta_{\mathrm{vg},l}$ (l=1,…,4) and $\beta_{\mathrm{gf},l}$ (l=1,2) are the coefficients of the covariable NDVI (average or all dates measurements) measured for line jth during several times at the vegetative ($x_{\mathrm{vg},jl}$) and grain filling ($x_{\mathrm{gf},jl}$) periods, respectively, and $\epsilon_{ij}$ are independent random errors assumed to be a normal variable with mean 0 and variance $\sigma^{2}$. Furthermore, it is assumed that the random effect of lines $g=(g_{1},\ldots ,g_{J}{)}^{T}$ is distributed as $N_{J}(0_{J},\sigma_{g}^{2}G)$, while the interaction year × line random effects $Yg=(\mathrm{year}\times g_{11},\ldots ,\mathrm{year}\times g_{1J},\ldots ,\mathrm{year}\times g_{21},\ldots ,\mathrm{year}\times g_{IJ}{)}^{T}$ follow a $N_{IJ}(0_{IJ},\sigma_{Yg}^{2}(I_{I}⊗G))$, with $0_{IJ}$ null vector of size I*J*, $I_{I}$ the identity matrix of dimensions I×I, ⊗ denote the Kronecker product, and G is the genomic relationship matrix (Van Raden 2008).

A Bayesian estimation of this model (GBLUP) was implemented with the BGLR R package using the defaults priors for the parameters (Pérez-Rodríguez and de los Campos 2014), where for both covariables ($x_{\mathrm{vg},jl}$ and $x_{\mathrm{gf},jl}$) a flat prior was used for its corresponding regression coefficients. This model was used in the evaluation of GP accuracy under the 5FCV, that is, the available data set was partitioned in five balanced sub-sets, and 4/5 of them were used to train the model, while the remaining (1/5, testing) were used to evaluate its performance. This process was repeated until each 1/5 part of the partition was left out of the training process to evaluate its performance, and finally, the average of the metric values obtained across all five partitions was reported as the global evaluation performance of the particular model being evaluated. This model was also used for the evaluation of the prediction performance of 1 entire year using all the lines from the other years (LOO); however, the terms $\mathrm{yea}r_{i}$ and $\mathrm{year}\times g_{ij}$ effects were removed.

#### Deep learning under the 5-fold cross-validation (5FCV)

**Fig. 1.**
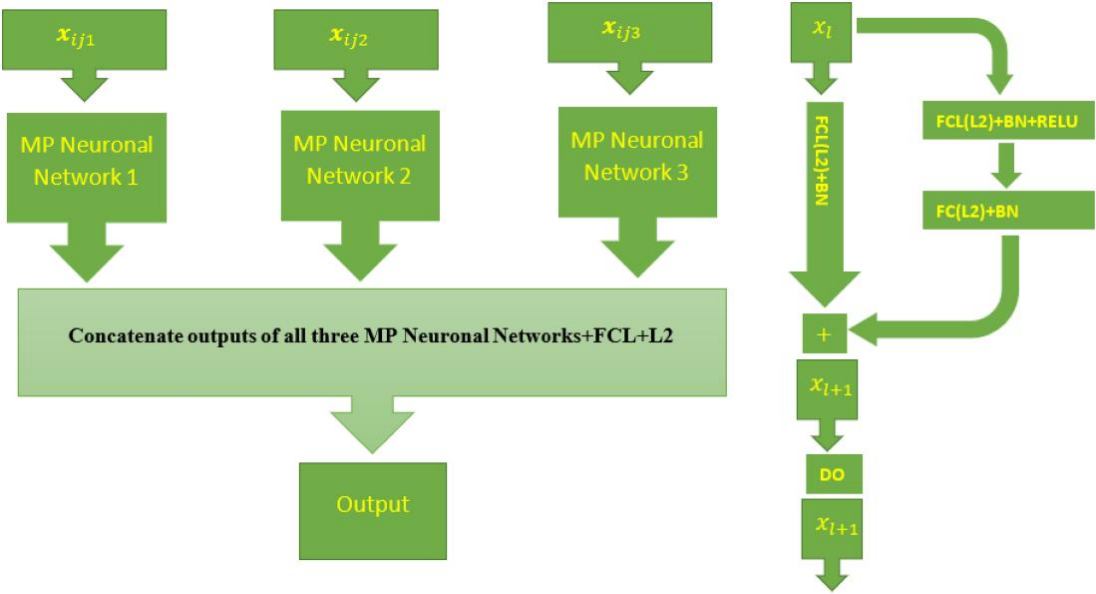
Left diagram: Multimodal deep learning model (MMDL) with 3 modalities (type of input). Right diagram: stacked residuals network (RestNet) composed of 2 sequence dense layers (FCL) applied in each MP neural network. FCL(L2)+ +BN + RELU denote the successive application of a full connected layer (FCL) with L2 regularization, batch normalization layer and relu activation. The meaning is similar for FCL(L2)+BN, while DO indicate the application of the dropout regularization. Batch Normalization (BN) also works as a regularizer to speed up the training process (He *et al*. 2016). To produce the final output, the concatenated outputs of three networks are used as an input in the output layer with 1 neuron, linear activation function, and L2 regularization for its weights (concatenate outputs of all 3 (multilayer perceptron deep neural network) MP neural networks + FCL + L2).

The same information previously described was used under the DL model, where genomic and NDVI covariables were measured in the 2 periods; VG and GF, were used as inputs. The DL model used for performing the GP accuracy under the 5FCV is a multimodal DL (MMDL; Ramachandram and Taylor 2017) with 3 modalities (type of information used; see Fig. 1) under multilayer perceptron deep neural network (MP) given by


(2) $$Y_{ij}=f(x_{ij};W)=f_{O}\left(b_{O}+\overset{3}{\underset{q=1}{\sum }}\;\overset{N_{n_{\mathrm{HL}}}^{\left(q\right)}}{\underset{k=1}{\sum }}\;w_{kq}^{\left(O\right)}x_{ijkq}^{\left(n_{\mathrm{HL}}^{\left(q\right)}\right)}\right)$$

where $x_{ij}=(x_{ij1}^{T},x_{ij2}^{T},x_{ij3}^{T}{)}^{T}$, $x_{ij1}^{T},x_{ij2}^{T}$, and $x_{ij3}^{T}$ are the 3-modality (type of inputs) vectors and are, respectively, the *ij* -th row of the following matrices, $X_{\mathrm{year}}$ is the matrix design of year, $Z_{L}^{*}=Z_{L}L^{T}$ with $Z_{L}$ the matrix design of lines and L is the upper triangular part of the Cholesky decomposition of $G(G=L^{T}L)$, $X_{\mathrm{NDVIs}}=[X_{\mathrm{vg}},X_{\mathrm{gf}}]$, and $X_{\mathrm{vg}}$ and $X_{\mathrm{gf}}$ are the matrix designs of NDVIs measurement in the vegetative and grain feeling periods, respectively. $n_{\mathrm{HL}}^{\left(q\right)}$ is the hidden layer for the *q* th type neural network created with modality *q*, $x_{ijq}^{T}$, q=1,2,3.

Additionally, $x_{ijkq}^{\left(l\right)}=f_{lq}\left(b_{klq}+\overset{N_{l-1}^{\left(q\right)}}{\underset{m=1}{\sum }}\;w_{kmq}^{\left(l\right)}x_{ijmq}^{\left(l-1\right)}\right),k=1,\ldots ,N_{l},$ are the hidden neurons in hidden layer *l*, $l=1,..,n_{\mathrm{HL}}^{\left(q\right)}$, for *q* th type neural network (modality) *q*, where;$x_{ijmq}^{\left(0\right)}=x_{ijmq},m=1,\ldots ,p_{q},$ are all the inputs (predictors) for the *q* th modality $x_{ijq}^{T}=(x_{ij1q},\ldots ,x_{ijp_{q}q})$, q=1,2,3; $b_{klq}$, $k=1,\ldots ,N_{l},$ are the bias for hidden neurons ($N_{l}^{\left(q\right)}$) in hidden layer *l* for the *q* th neural network of modality *q* (NN), $w_{kmq}^{\left(l\right)},$$m=1,\ldots ,N_{l-1}^{\left(q\right)}$, are the weights to compute the *k* -th neuron in hidden layer *l* from the $N_{l-1}^{\left(q\right)}$ neurons of layer l−1 for the *q* th type neural network (modality) *q*, $b_{O},w_{kq}^{\left(O\right)}\mathrm{and}$$f_{O}$ are the bias, weights, and the activation function for the output layer, respectively; while $f_{lq}$, $l=1,\ldots ,n_{\mathrm{HL}}^{\left(q\right)},$ are the activations functions for all the hidden layers in the *q*th modality; and W is the vector of all weights in the model. After each dense layer and before activation, a batch normalization layer was applied to approximately standardized (mean close to 0 and standard deviation close to 1) corresponding outputs (see more details in Fig. 1).

#### Deep learning under leave-one-year out (LOO)

To evaluate DL models for predicting the performance of 1 entire year using all the lines from the other year, the same model (2) was used; however in this version only 2 modalities were used: $Z_{L}^{*}$ and $X_{\mathrm{NVIs}}.$ All DL models applied are versions of model (2) with a stacked residuals network (RestNet) composed of 2 sequence layers (He *et al*. 2016) and implemented with library TensorFlow in Python software. The model was fitted using a Batch_size value equal to 32, 128 epochs and the Adam optimizer (a popular stochastic gradient descend method to minimize the penalized loss function in a DL), and using callback options of the fit keras function. We specified an adaptative exponential decay learning scheduler.

For the training process, we established the validation split argument equal to 0.10 and again by means of the callback option, an early stopping rule was stated with monitor = “loss,” mode = “min,” and patience= “Pat” to check if the loss function in training data at the end of each epoch ceases to decrease, and if it continues to an additional Pat epoch, the training is stopped. Likewise, to prevent overfitting, dropout and L2 regularization were added at each layer involved, except for the output layer, where only the last one was applied. Learning decay (wd), patience values (Pat), dropout rate (DO), and regularization parameters (λ) were also considered as hyperparameters to be tuned. L2 regularization penalized the loss function (sum of squared error loss, for example) with the sum of squared weights by means of the regularization parameter (λ) that controls the degree to which the weights are shrunk toward zero, reducing the complexity of the model and avoiding fitting the training data too well. Dropout consists of setting a random fraction of the weights to 0 at each step during training time.

In all, for each modality (type of input) under an MP, the number of units after the second hidden layer was equal to half of the units in the preceding layer, that is, if $N_{1}^{\left(q\right)}$ is the number of units of the first hidden layer in the NN *q*, then the units for the rest of the layers was taken as $N_{l}^{\left(q\right)}=N_{1}^{\left(q\right)}/2^{\left(l-1\right)},l=2,\ldots ,n_{\mathrm{HL}}^{\left(q\right)}$, where the *x* denotes the functions that take the largest integer less than *x*. Additionally, in all hidden layers the rectifier linear unit (relu) activation function was used, except for the output layer because the traits analyzed were quantitative with an approximante normal distribution and a linear activation was applied.

The hyperparameters of the MMDL model were tuned using the library bayes_opt with 150 iterations, where the objective was to find the “optimal” hyperparameter values that minimize the validation means squared error. The complete list of hyperparameters and its domain space where the “optimal” values were searched is shown in Table 1. Further references in the manuscript will use MMDL as the specific DL when only 5FCV was used in DS1. The models were run in 1 computer node with 16 GB of RAM and 8 cores, using Python version 3.8.0 and TensorFlow 2.7.0, requiring an average of 8 hours to train a model with the specified characteristics described in the paper.

**Table 1.** Hyperparameters of DL model 2 and its domain space.

Hyperparameter	Hyperparameter	Bounds
Hidden layers	$n_{\mathrm{HL}}^{\left(1\right)}$	(1,4)
	$n_{\mathrm{HL}}^{\left(2\right)}$	(1,6)
	$n_{HL}^{\left(3\right)}$	(1,6)
Number of neurons	$N_{l}^{\left(1\right)}$	0,16)
	$N_{l}^{\left(2\right)}$	0,1024)
	$N_{l}^{\left(3\right)}$	0,64)
Regularization parameter for L2	λ	(1e-8,1e-2)
Dropout	DO	($1\times 10^{-4}$,0.5)
Log weight decay	lwd=ln(wd)	$\left(\ln\;(4\times 10^{-5}),\ln\;(4\times 10^{-1})\right)$
Patience	Pat	(0,64)
Log learning rate	llr=ln(lr)	$\left(\ln\;(1\times 10^{-5}),\ln\;(1\times 10^{-2})\right)$

#### Gradient boosting machine (GBM) with LOO cross-validation

The GBM is a machine learning algorithm originally proposed for improving weak classifiers, that can be used with many statistical learning methods including regression (Hastie *et al*. 2009). It is a modification of the original boosting algorithm and was introduced by Friedman (2001), proposing to fit a base learner on a subsample of the training set drawn at random without replacement at each iteration of the algorithm.

We implemented the following GBM algorithm proposed by Friedman (2001), where $\left(y_{i},x_{i}\right)$, for i=1,2,…,n are the data (output and input), *M* number of iterations, φ(y,f) is the chosen loss function, and choose the base-learner model h(x,θ):

Algorithm: initialize $f_{0}$ with a constant and for t=1 to *M*, repeat Steps 1–4:

Step 1: compute the negative gradient of $\varphi (y_{i},f)$ with respect to *f* ($g_{t}(x_{i})$)

Step 2: fit a new base-learner function $h(x,\theta_{t})$ for predicting $g_{t}(x_{i})$ from the covariables $x_{i}$.

Step 3: find the best gradient descent step-size $\rho_{t}$:


$$\rho_{t}=\arg\;\underset{\rho }{\min}\;\overset{n}{\underset{i=1}{\sum }}\;\varphi [y_{i},{\hat{f}}_{t-1}(x_{i})+\rho h(x_{i},\theta_{t})]$$

Step 4: update the function estimate: ${\hat{f}}_{t}\leftarrow {\hat{f}}_{t-1}+\rho_{t}h(x,\theta_{t})$

Step 5: final predictions: $\hat{f}(x)={\hat{f}}_{M}(x)$

The GBM method was implemented using a Gaussian loss function (based on normal distribution), and as a base-learner model ($h(x,\theta_{t})$), the decision trees were used with interaction depth equal to 1, number of trees equal to 5000, the shrinkage value 0.001, with the minimum number of observations in the terminal nodes of the tree equal to 10, and 0.5 as the fraction of the training set observations randomly selected to propose the next tree in the expansion. This was done using the GBM R package (Greenwell *et al*. 2022). Like in the GBLUP and DL models, the information used here as inputs contained information of environments, the information of the genotypes with markers and the information of NDVIs measurements for the 5FCV evaluation. The LOO evaluation was similar.

#### Support vector regression (SVR) with LOO cross-validation

Support vector regression is an extension of support vector machine developed for classification and was proposed by Drucker *et al*. (1996) as a scheme to predict continuous values. Support vector regression in linear case seeks beta coefficients that minimize the regularized sum of the deviation error not larger than some positive constant (ϵ) defined as


$$L(\beta )=C\overset{n}{\underset{i=1}{\sum }}\;V_{\epsilon }(y_{i}-\beta_{0}-x_{i}^{T}\beta_{0})+\frac{1}{2}\left(\overset{p}{\underset{\;j=1}{\sum }}\;\beta_{j}^{2}\right)$$

where $L_{\epsilon }(e)=0$ if e<ϵ and $L_{\epsilon }(e)=e-\epsilon$ otherwise and *C* (cost constrain violations) is the inverse of the regularization parameter. Equivalently, SVR solve the following optimization problem (in case this is feasible):


$$\mathrm{Minimize}\;\frac{1}{2}|\left|\beta_{0}\right|{|}^{2}$$

$$\mathrm{subject}\;\mathrm{to}\;|y_{i}-\beta_{0}-x_{i}^{T}\beta_{0}|\leq \epsilon ,\;i=1,\ldots ,n.$$

When this problem does not have a solution (is infeasible), slack variables are usually introduced to allow for some errors and obtain a less rigid model, allowing an ϵ -precision approximation for some of the training data points. For more specific details about this extended formulation, see Smola and Schölkopf (2004). This technique was implemented with the R package e1071 (Meyer *et al*. 2022), using linear kernel and default values for the rest of the parameters (ϵ = 0.1 in the insensitive loss-function, tolerance 0.01, and cost of constraint violations equal to 1).

## Result

**Fig. 2.**
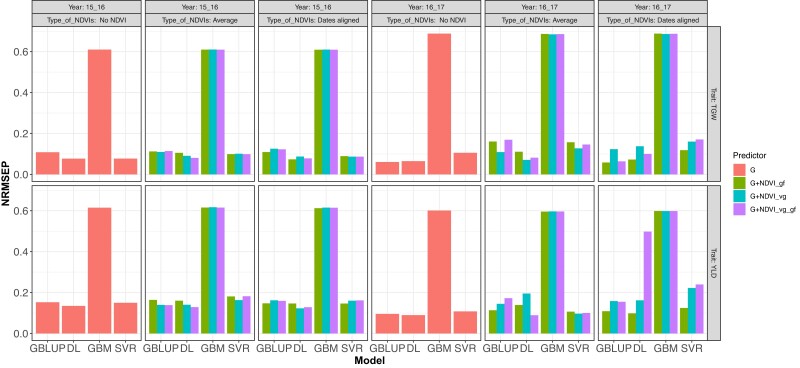
DS1.Normalized Root Mean Squared Error of Prediction (NRMSEP) for the prediction of a complete year using information from the other year (leave one environment out, LOO) for traits TGW and YLD using the models fitted with GBLUP with BGLR (R software) and with DL (Python software) with the predictors G (red Type of NDVI__, no use of NDVI), which correspond to the genomic matrix, G + NDVIs_gf (green), G + NDVIs_vg (blue), and G + NDVIs_vg_gf (purple) for the NDVIs covariate in any of its 2 types NDVI averages and dates aligned.

**Fig. 3.**
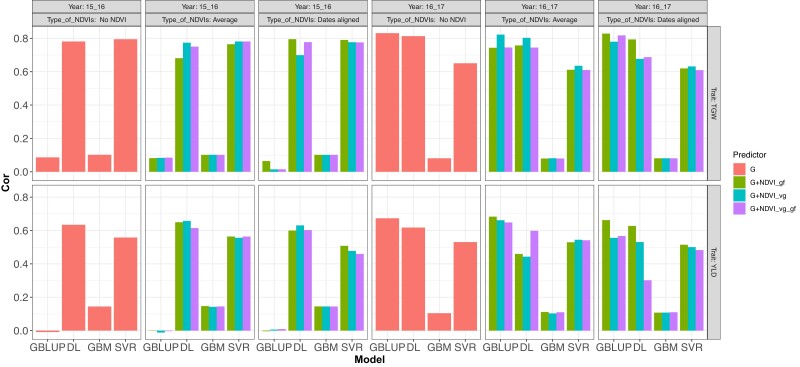
DS1.Correlation Mean (Cor Mean) for the prediction of a complete year using information from the other year (leave one environment out, LOO) for traits TGW and YLD using the models fitted GBLUP with BGLR (R software) and with DL (Python software) with the predictors G (red Type of NDVI__, no use of NDVI), which correspond to the genomic matrix, G + NDVIs_gf (green), G + NDVIs_vg (blue), and G + NDVIs_vg_gf (purple) for the NDVIs covariate in any of its 2 types NDVI averages and dates aligned.

**Fig. 4.**
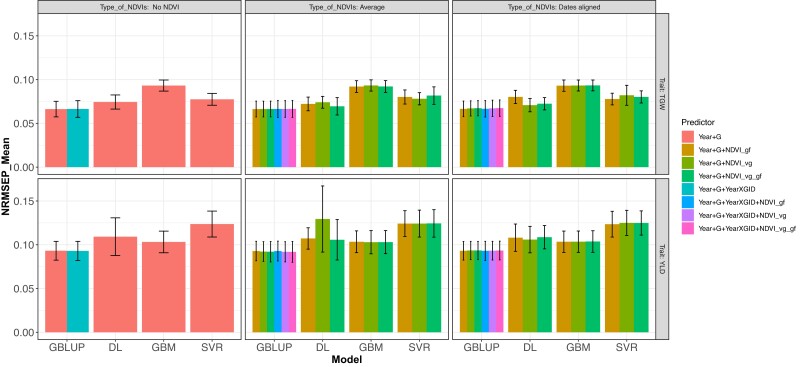
DS1. Normalized Root Mean Squared Error of Prediction (NRMSEP) under a 5-fold cross-validation for traits TGW and YLD, for models fitted under BGLR (R software) and multimodal DL (Python software) with the predictors being year + G, which correspond to the year and the genomic matrix, year + G + NVVDIs_gf, and year + G + NDVIs_vg, adding the covariate NDVIs as average or aligned dates (Type_NDVIs). For the GBLUP the following predictors are also used, year + G + year × G, making use of the interaction between the genomic matrix and the year, year + G + year × GID + NDVIs_gf, year + GID + year × GID + NDVIs_vg, and year + G + year × GID + NDVIs_vg_gf that adds the NDVIs covariate in 1 of its 2 types (Type of NDVIs), averages and dates aligned.

**Fig. 5.**
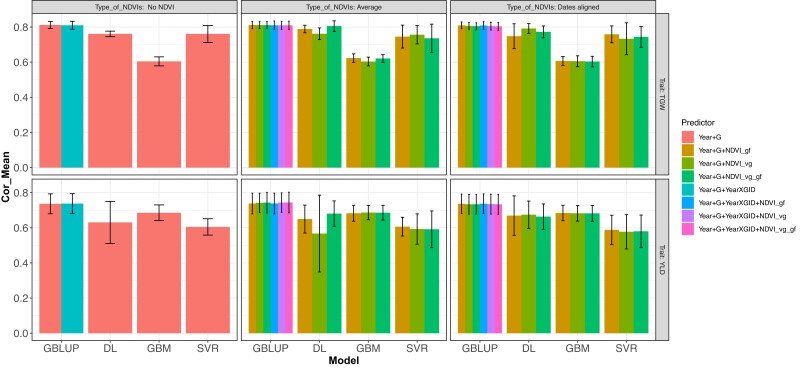
DS1. Correlation Mean (Cor Mean) under a 5-fold cross-validation for the prediction of traits TGW and YLD, for models fitted under GBLUP with BGLR (R software) and multimodal DL (Python software) with the predictors being year + G, which corresponds to the year and the genomic matrix, year + G + NVVDIs_gf, and year + G + NDVIs_vg, adding the covariate NDVIs as average or dates aligned (Type of NDVIs). For the GBLUP the following predictors are also used, year + G + year × G, making use of the interaction between the genomic matrix and the year, year + G + year × GID + NDVIs_gf, year + GID + year × GID + NDVIs_vg, and year + G + year × GID + NDVIs_vg_gf that adds the NDVIs covariate in 1 of its 2 types (Type of NDVIs), averages and dates aligned.

**Fig. 6.**
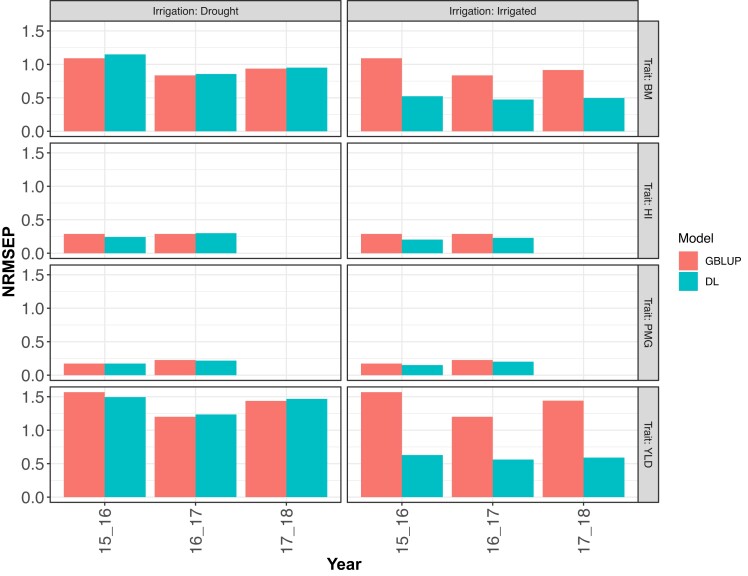
DS2. Normalized Root Mean Squared Error of Prediction (NRMSEP) for the prediction of a trait (BM, HI, PMG, and YLD) in drought (irrigated) environment using information from an irrigated (drought) environment (leave one environment out, LOO) using a sub-model of (1) fitted with GBLUP and with a sub-model of DL model (2), both with the predictor G. Each of these sub-models were fitted for each year (2015–2016, 2016–2017, and 2017–2018) and each trait.

**Fig. 7.**
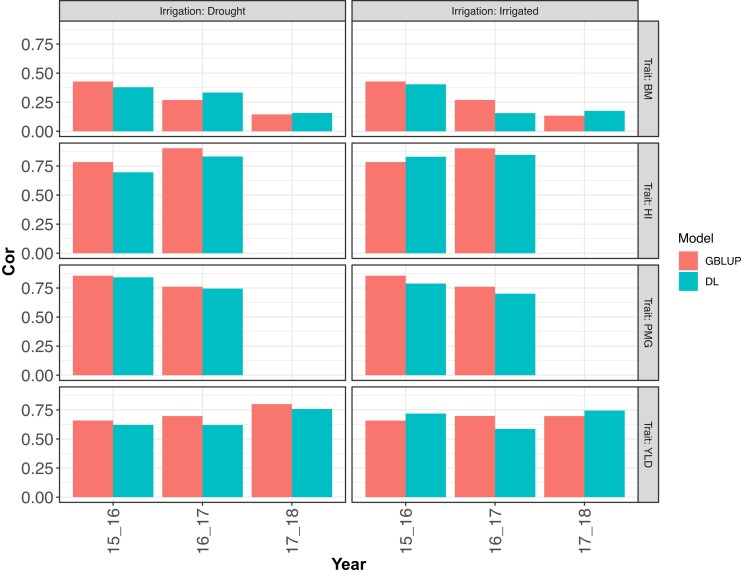
DS2. Correlation Mean (Cor Mean) for the prediction of a trait (BM, HI, PMG, and YLD) in drought (irrigated) environment using information from an irrigated (drought) environment (leave one environment out, LOO) using a sub-model of 1 fitted with GBLUP and with a sub-model of DL model 2, both with the predictor G. Each of these sub-models were fitted for each year (2015–2016, 2016–2017, and 2017–2018) and each trait.

As already mentioned, DS1 was fitted with models GBLUP, DL, GBM, and SVR. The genomic prediction ability assessment was performed using 5FCV and LOO with genomic and image information. Furthermore, all tables (Tables 1–10) and Figs. 1–5 show results based on DS1. The multimodal DL applies only to the 5FCV validation of DS1 (Figs. 4 and 5). DS2 was fitted with models GBLUP and DL; the genomic prediction ability assessment was performed using LOO with only genomic data. Results from DS2 are displayed in Figs. 6 and 7.

### DS1

Several models were fitted for 2 traits (YLD and TGW); for different predictors; training sets (5FCV and LOO) including genomic (G), year, G × year; and the different types of NDVIs (NDVI average across dates or NDVI aligned for dates), using the GBLUP with BGLR (R software) and DL (Python software). We considered fitting models with only the genomic component (G) (with no NDVI included in the model) and models that included NDVI as covariables measured at the vegetative (NDVI_vg) and grain filing stage (NDVI_gf), included both vegetative stage and grain filing (NDVI_vg_gf). Each of these cases were tested under 2 different schemes: NDVI with dates aligned and NDVI with average values across all data.

The different genomic prediction accuracies for the 5FCV obtained from fitted sub-models of 1 and 2 are shown in Tables 2 and 3. In the last 2 blocks of Table 3 (GBM-TGW and GBM-YLD) and Table 4, prediction accuracies are shown under the same 5FCV strategy for 2 additional explored machine learning models, gradient boosting machine (GBM) and support vector regression (SVR). The first method was implemented using the R packages gbm (Greenwell *et al*. 2022) with 5,000 trees and default values for interaction depth and learning rate (1 and 0.001); the second one was implemented with the R package e1071 (Meyer *et al*. 2022) using linear kernel and default values for the rest of the parameters (ϵ = 0.1 in the insensitive loss-function, tolerance 0.01, and cost of constraint violations equal to 1).

**Table 2.** DS1.

Model	NRMSEP	Cor	Type of NDVIs
**GBLUP-TGW**			
Year + G	0.0663 (0.0089)	0.8116 (0.0195)	—
Year + G + NDVI_gf	0.0664 (0.0091)	0.8112 (0.0209)	Average
Year + G + NDVI_gf	0.0666 (0.0089)	0.8095 (0.0198)	Dates aligned
Year + G + NDVI_vg	0.0664 (0.009)	0.8111 (0.0199)	Average
Year + G + NDVI_vg	0.0671 (0.0087)	0.8064 (0.0189)	Dates aligned
Year + G + NDVI_vg_gf	0.0664 (0.009)	0.8108 (0.0202)	Average
Year + G + NDVI_vg_gf	0.0673 (0.0087)	0.8047 (0.0196)	Dates aligned
Year + G + year × GID	0.0665 (0.0095)	0.81 (0.0227)	—
Year + G + year × GID + NDVI_gf	0.0665 (0.0097)	0.81 (0.0241)	Average
Year + G + year × GID + NDVI_gf	0.0667 (0.0094)	0.8086 (0.0223)	Dates aligned
Year + G + year × GID + NDVI_vg	0.0665 (0.0095)	0.81 (0.0229)	Average
Year + G + year × GID + NDVI_vg	0.0672 (0.0094)	0.8056 (0.0222)	Dates aligned
Year + G + year × GID + NDVI_vg_gf	0.0665 (0.0097)	0.8097 (0.0239)	Average
Year + G + year × GID + NDVI_vg_gf	0.0674 (0.0094)	0.8039 (0.0223)	Dates aligned
**GBLUP–YLD**			
Year + G	0.0932 (0.0107)	0.7364 (0.0567)	—
Year + G + NDVI_gf	0.0929 (0.0112)	0.7382 (0.0593)	Average
Year + G + NDVI_gf	0.0931 (0.0106)	0.7365 (0.0555)	Dates aligned
Year + G + NDVI_vg	0.0922 (0.0113)	0.7421 (0.0553)	Average
Year + G + NDVI_vg	0.0935 (0.0104)	0.734 (0.0563)	Dates aligned
Year + G + NDVI_vg_gf	0.0921 (0.0117)	0.7431 (0.0588)	Average
Year + G + NDVI_vg_gf	0.0935 (0.0104)	0.7338 (0.0553)	Dates aligned
Year + G + year × GID	0.093 (0.0109)	0.7375 (0.0568)	—
Year + G + year × GID + NDVI_gf	0.0928 (0.0114)	0.7382 (0.059)	Average
Year + G + year × GID + NDVI_gf	0.0931 (0.0109)	0.7367 (0.0563)	Dates aligned
Year + G + year × GID + NDVI_vg	0.092 (0.0115)	0.7436 (0.0556)	Average
Year + G + year × GID + NDVI_vg	0.0933 (0.0107)	0.7348 (0.0565)	Dates aligned
Year + G + year × GID + NDVI_vg_gf	0.0919 (0.0119)	0.7439 (0.0589)	Average
Year + G + year × GID + NDVI_vg_gf	0.0934 (0.0107)	0.7337 (0.0567)	Dates aligned

Results of 5-fold cross-validation (5FCV) for 2 traits TGW (thousand grain weight) and YLD (grain yield) for 2 metrics, Normalized Root Mean Squares Error Prediction [NRMSEP (SD)] and correlation [Cor (SD)] between observed and predicted values for models including year (year), genomic (G), interaction year and genomic (year × G), and NDVI. Two types of NDVIs were included for NDVI vegetative (vg) and NDVI grain filling (gf), one aligning the dates (dates aligned) and the other one averaging (average) the dates. SD is the standard deviation across folds. Fitted sub-models of model 1 (GBLUP) with software BGLR.

**Table 3.** DS1.

Model	NRMSEP	Correlation	Type of NDVIs
**MMDL-TGW**			
Year + G	0.0744 (0.0081)	0.7608 (0.0158)	—
Year + G + NDVI_gf	0.0722 (0.0078)	0.7888 (0.0215)	Average
Year + G + NDVI_gf	0.0802 (0.0076)	0.7478 (0.0707)	Dates aligned
Year + G + NDVI_vg	0.0741 (0.0067)	0.7617 (0.0333)	Average
Year + G + NDVI_vg	0.0708 (0.0076)	0.7917 (0.0277)	Dates aligned
Year + G + NDVI_vg_gf	0.0695 (0.0099)	0.8054 (0.03)	Average
Year + G + NDVI_vg_gf	0.0724 (0.007)	0.7726 (0.0336)	Dates aligned
**MMDL-YLD**			
Year + G	0.1092 (0.0215)	0.6305 (0.1198)	—
Year + G + NDVI_gf	0.1072 (0.0122)	0.6498 (0.0794)	Average
Year + G + NDVI_gf	0.1081 (0.0156)	0.6693 (0.1124)	Dates aligned
Year + G + NDVI_vg	0.1294 (0.0378)	0.567 (0.2188)	Average
Year + G + NDVI_vg	0.1059 (0.0151)	0.6746 (0.0777)	Dates aligned
Year + G + NDVI_vg_gf	0.1057 (0.0231)	0.6809 (0.0725)	Average
Year + G + NDVI_vg_gf	0.1087 (0.0133)	0.6636 (0.0721)	Dates aligned
**GBM-TGW**			
Year + G	0.0932 (0.0063)	0.6046 (0.0256)	—
Year + G + NDVI_gf	0.092 (0.0067)	0.6233 (0.0241)	Average
Year + G + NDVI_gf	0.0931 (0.0064)	0.6068 (0.0256)	Dates aligned
Year + G + NDVI_vg	0.0933 (0.0064)	0.6037 (0.025)	Average
Year + G + NDVI_vg	0.0933 (0.0063)	0.6056 (0.0306)	Dates aligned
Year + G + NDVI_vg_gf	0.0921 (0.0067)	0.6211 (0.0221)	Average
Year + G + NDVI_vg_gf	0.0933 (0.0062)	0.6038 (0.0301)	Dates aligned
**GBM-YLD**			
Year + G	0.1033 (0.0124)	0.6854 (0.0448)	—
Year + G + NDVI_gf	0.1034 (0.0125)	0.6829 (0.0454)	Average
Year + G + NDVI_gf	0.1034 (0.0122)	0.6842 (0.0439)	Dates aligned
Year + G + NDVI_vg	0.1029 (0.0133)	0.6869 (0.041)	Average
Year + G + NDVI_vg	0.1035 (0.0122)	0.6826 (0.0444)	Dates aligned
Year + G + NDVI_vg_gf	0.1030 (0.0132)	0.686 (0.0421)	Average
Year + G + NDVI_vg_gf	0.1037 (0.0123)	0.6818 (0.0452)	Dates aligned

Results of 5-fold cross-validation (5FCV) for 2 traits TGW (thousand grain weight) and YLD (grain yield) for 2 metrics, Normalized Root Mean Squares Error Prediction [NRMSEP (SD)] and correlation [Cor (SD)] between observed and predicted values for models including year (year), genomic (G), and NDVI. Two types of NDVIs were included for NDVI vegetative (vg) and NDVI grain filling (gf), one aligning the dates (dates aligned) and the other one averaging (average) the dates. SD is the standard deviation across folds. Different fitted sub-models of multimodal DL 2, first 2 blocks in the table (MMDL-TGW and MMDL-YLD). The second 2 blocks (GBM-TGW and GBM-YLD) correspond to the performance of the gradient boosting machine (GBM) by using 5,000 trees, interaction depth and multimodal deep learning (MMDL) rate the default values (1 and 0.001) in the gb R package.

**Table 4.** DS1.

Model	NRMSEP	Correlation	Type of NDVIs
**SVR-TGW**			
Year + G	0.0775 (0.0068)	0.7608 (0.0484)	—
Year + G + NDVI_gf	0.0801 (0.0081)	0.7456 (0.0652)	Average
Year + G + NDVI_gf	0.0778 (0.0067)	0.7585 (0.048)	Dates aligned
Year + G + NDVI_vg	0.0781 (0.007)	0.7564 (0.0524)	Average
Year + G + NDVI_vg	0.0821 (0.0114)	0.7333 (0.0907)	Dates aligned
Year + G + NDVI_vg_gf	0.0817 (0.01)	0.7358 (0.0809)	Average
Year + G + NDVI_vg_gf	0.0802 (0.0069)	0.7441 (0.0598)	Dates aligned
**SVR-YLD**			
Year + G	0.1237 (0.0148)	0.6049 (0.0466)	—
Year + G + NDVI_gf	0.1242 (0.0147)	0.6065 (0.0533)	Average
Year + G + NDVI_gf	0.1235 (0.0147)	0.588 (0.0838)	Dates aligned
Year + G + NDVI_vg	0.1242 (0.0154)	0.5928 (0.0863)	Average
Year + G + NDVI_vg	0.125 (0.0144)	0.5772 (0.0974)	Dates aligned
Year + G + NDVI_vg_gf	0.1244 (0.0157)	0.5915 (0.1043)	Average
Year + G + NDVI_vg_gf	0.1249 (0.0138)	0.5798 (0.0929)	Dates aligned

Results of 5-fold cross-validation (5FCV) for 2 traits TGW (thousand grain weight) and YLD (grain yield) for 2 metrics, Normalized Root Mean Squares Error Prediction [NRMSEP (SD)] and correlation [Cor (SD)] between observed and predicted values for models including year (year), genomic (G), and NDVI. Two types of NDVIs were included for NDVI vegetative (vg) and NDVI grain filling (gf), one aligning the dates (dates aligned) and the other one averaging (average) the dates. SD is the standard deviation across folds. Performance prediction of support vector regression (SVR) by using linear kernel and default values for the rest of the parameters as done with the e1071 R package (ϵ=0.1 in the insensitive loss-function, tolerance 0.01, and cost of constraint violations equal to 1).

Trait TGW had GP accuracies higher than trait YLD, and the mean correlation between observed and predicted values (Cor Mean) was consistently higher (and NRMSEP consistently smaller) for NDVI average than NDVI with data aligned (Table 2). Moreover, the GE model did not improve GP accuracy nor did the inclusion of various NDVIs (NDVI_vg, NDVI_gy, and NDVI_vg_gf) except for NDVI_average. Similar result patterns were found for DL (first two blocks in Table 3, TGW-DL, and YLD-DL); however, GP accuracy was slightly lower than those achieved by the models fitted by GBLUP. Note that the term year × G was not included in DL, as the previous results indicated (Montesinos-López *et al*. 2018, 2019) that including GE reduced the GP accuracy of DL.

Furthermore, the results (Table 3) of the GBM in trait TGW show lower accuracy with both metrics when compared to what was achieved by the DL model, while with the trait YLD, DL show a slightly lower accuracy in almost all the cases except with the predictors year + G + NDVI_gf and year + G + NDVI_vg_gf when average NDVI was used. Now, with respect to the SVR (Table 4), the results found for DL with metric Cor Mean were slightly better than SVR except in trait TGW with the models that included aligned NDVIs in its predictors. Here, the differences in the results were moderately better for DL. Similar results were found with metric NRMSEP, but for trait YLD, the difference was more remarkable and favored DL except in the case of predictor year + G + NDV_vg, where the difference was almost indistinguishable.

The GP accuracy for the LOO prediction including the various NDVIs data when models were fitted with the BGLR R package, model 1 and the DL approach are shown in Tables 5 and 6; the corresponding results for GBM and SVR are listed in Tables 7 and 8. Under sub-models 1, for both traits and for all cases of NDVI, dates aligned and the average prediction of cycle (year) 2016–2017 using cycle 2015–2016 as training were much higher than those obtained when predicting 2015–2016 using 2016–2017 as training (Table 5); these results are for both metrics Cor Mean and NRMSEP. This does not occur when using DL (Table 6) where model prediction includes NDVI Dates Aligned versus Average. With DL the GP accuracy is slightly lower than those found when fitting models with GBLUP for both traits.

**Table 5.** DS1.

Year	Predictor	NRMSEP	Correlation	Type of NDVIs
**GBLUP-TGW**				
2015–2016	G	0.1085	0.0865	—
2016–2017	G	0.0607	0.831	—
2015–2016	G + NDVI_gf	0.1124	0.0819	Average
2015–2016	G + NDVI_gf	0.1093	0.0646	Dates aligned
2016–2017	G + NDVI_gf	0.1608	0.7433	Average
2016–2017	G + NDVI_gf	0.0583	0.8284	Dates aligned
2015–2016	G + NDVI_vg	0.1095	0.0833	Average
2015–2016	G + NDVI_vg	0.1256	0.0144	Dates aligned
2016–2017	G + NDVI_vg	0.1091	0.8224	Average
2016–2017	G + NDVI_vg	0.1234	0.7798	Dates aligned
2015–2016	G + NDVI_vg_gf	0.1142	0.0848	Average
2015–2016	G + NDVI_vg_gf	0.1227	0.0151	Dates aligned
2016–2017	G + NDVI_vg_gf	0.1694	0.745	Average
2016–2017	G + NDVI_vg_gf	0.0639	0.8175	Dates aligned
**GBLUP-YLD**				
2015–2016	G	0.1527	−0.0085	—
2016–2017	G	0.0958	0.6731	—
2015–2016	G + NDVI_gf	0.1641	−0.0015	Average
2015–2016	G + NDVI_gf	0.1473	−0.0038	Dates aligned
2016–2017	G + NDVI_gf	0.1136	0.6831	Average
2016–2017	G + NDVI_gf	0.109	0.6622	Dates aligned
2015–2016	G + NDVI_vg	0.1398	−0.0117	Average
2015–2016	G + NDVI_vg	0.1622	0.0061	Dates aligned
2016–2017	G + NDVI_vg	0.145	0.6613	Average
2016–2017	G + NDVI_vg	0.1587	0.5558	Dates aligned
2015–2016	G + NDVI_vg_gf	0.1386	−0.0033	Average
2015–2016	G + NDVI_vg_gf	0.1593	0.009	Dates aligned
2016–2017	G + NDVI_vg_gf	0.1726	0.6484	Average
2016–2017	G + NDVI_vg_gf	0.155	0.5671	Dates aligned

Results of predicting 1 entire year using the other (leave one environment out, LOO) for 2 traits TGW (thousand grains weight) and YLD (grain yield) for 2 metrics Normalized Root Mean Squares Error Prediction (NRMSEP) and correlation (Cor Mean) between observed and predicted values for models including and genomic (G) and NDVI. Two types of NDVIs were included for NDVI vegetative (vg) and NDVI grain filling (gf), one aligning the dates (dates aligned) and the other one averaging (average) the dates. Fitted sub-models of model 1 with (GBLUP) BGLR R package.

**Table 6.** DS1.

Year	Predictor	NRMSEP	Correlation	Type of NDVIs
**DL-TGW**				
2015–2016	G	0.0648	0.813	—
2016–2017	G	0.0771	0.781	—
2015–2016	G + NDVI_gf	0.1109	0.7574	Average
2015–2016	G + NDVI_gf	0.0728	0.7932	Dates aligned
2016–2017	G + NDVI_gf	0.1055	0.681	Average
2016–2017	G + NDVI_gf	0.0737	0.7948	Dates aligned
2015–2016	G + NDVI_vg	0.0709	0.8027	Average
2015–2016	G + NDVI_vg	0.1375	0.6767	Dates aligned
2016–2017	G + NDVI_vg	0.0907	0.7739	Average
2016–2017	G + NDVI_vg	0.0875	0.6989	Dates aligned
2015–2016	G + NDVI_vg_gf	0.0817	0.7443	Average
2015–2016	G + NDVI_vg_gf	0.1002	0.6872	Dates aligned
2016–2017	G + NDVI_vg_gf	0.0807	0.7501	Average
2016–2017	G + NDVI_vg_gf	0.0784	0.7771	Dates aligned
**DL-YLD**				
2015–2016	G	0.0899	0.6178	—
2016–2017	G	0.1349	0.6341	—
2015–2016	G + NDVI_gf	0.1393	0.4596	Average
2015–2016	G + NDVI_gf	0.0985	0.6273	Dates aligned
2016–2017	G + NDVI_gf	0.1604	0.6494	Average
2016–2017	G + NDVI_gf	0.1465	0.5998	Dates aligned
2015–2016	G + NDVI_vg	0.1952	0.4431	Average
2015–2016	G + NDVI_vg	0.1619	0.5307	Dates aligned
2016–2017	G + NDVI_vg	0.1404	0.6572	Average
2016–2017	G + NDVI_vg	0.1229	0.6305	Dates aligned
2015–2016	G + NDVI_vg_gf	0.0893	0.5979	Average
2015–2016	G + NDVI_vg_gf	0.4979	0.3018	Dates aligned
2016–2017	G + NDVI_vg_gf	0.1289	0.6146	Average
2016–2017	G + NDVI_vg_gf	0.1285	0.6024	Dates aligned

Results of predicting 1 entire year using the other (leave one environment out, LOO) for 2 traits TGW (thousand grain weight) and YLD (grain yield) for 2 metrics, Normalized Root Mean Squares Error Prediction (NRMSEP) and correlation (Cor Mean) between observed and predicted values for models including and genomic (G) and NDVI. Two types of NDVIs were included for NDVI vegetative (vg) and NDVI grain filling (gf), one aligning the dates (dates aligned) and the other one averaging the dates (average). (SD standard deviation). Fitted sub-models of DL model 2.

**Table 7.** DS1.

Year	Predictor	NRMSEP	Correlation	Type of NDVIs
**GBM-TGW**				
2015–2016	G	0.61	0.1018	—
2016–2017	G	0.6881	0.0809	—
2015–2016	G + NDVI_gf	0.6098	0.1017	Average
2015–2016	G + NDVI_gf	0.6093	0.1016	Dates aligned
2016–2017	G + NDVI_gf	0.6862	0.0795	Average
2016–2017	G + NDVI_gf	0.6881	0.081	Dates aligned
2015–2016	G + NDVI_vg	0.6102	0.1018	Average
2015–2016	G + NDVI_vg	0.6101	0.1018	Dates aligned
2016–2017	G + NDVI_vg	0.6843	0.0812	Average
2016–2017	G + NDVI_vg	0.686	0.0809	Dates aligned
2015–2016	G + NDVI_vg_gf	0.6095	0.1016	Average
2015–2016	G + NDVI_vg_gf	0.6094	0.1016	Dates aligned
2016–2017	G + NDVI_vg_gf	0.6857	0.0795	Average
2016–2017	G + NDVI_vg_gf	0.6871	0.0808	Dates aligned
**GBM-YLD**				
2015–2016	G	0.6148	0.1444	—
2016–2017	G	0.6011	0.1044	—
2015–2016	G + NDVI_gf	0.6154	0.1468	Average
2015–2016	G + NDVI_gf	0.6123	0.1447	Dates aligned
2016–2017	G + NDVI_gf	0.5957	0.1117	Average
2016–2017	G + NDVI_gf	0.5986	0.1075	Dates aligned
2015–2016	G + NDVI_vg	0.6168	0.1423	Average
2015–2016	G + NDVI_vg	0.6149	0.1446	Dates aligned
2016–2017	G + NDVI_vg	0.5967	0.103	Average
2016–2017	G + NDVI_vg	0.598	0.1078	Dates aligned
2015–2016	G + NDVI_vg_gf	0.6152	0.1447	Average
2015–2016	G + NDVI_vg_gf	0.6145	0.1444	Dates aligned
2016–2017	G + NDVI_vg_gf	0.5961	0.11	Average
2016–2017	G + NDVI_vg_gf	0.5981	0.11	Dates aligned

Results of predicting 1 entire year using the other (leave one environment out, LOO) for 2 traits TGW (thousand grains weight) and YLD (grain yield) for 2 metrics, Normalized Root Mean Squares Error Prediction (NRMSEP) and correlation (Cor Mean) between observed and predicted values for models including and genomic (G) and NDVI. Two types of NDVIs were included for NDVI vegetative (vg) and NDVI grain filling (gf), one aligning the dates (Dates aligned) and the other one averaging (average) the dates. Gradient boosting machine (GBM) by using 5,000 trees, with interaction depth and learning rate the default values (1 and 0.001) in the gbm R package.

**Table 8.** DS1.

Year	Predictor	NRMSEP	Correlation	Type of NDVIs
**SVR-TGW**				
2015–2016	G	0.0773	0.7945	—
2016–2017	G	0.1057	0.6503	—
2015–2016	G + NDVI_gf	0.0992	0.7646	Average
2015–2016	G + NDVI_gf	0.0892	0.7901	Dates aligned
2016–2017	G + NDVI_gf	0.157	0.6115	Average
2016–2017	G + NDVI_gf	0.1184	0.6196	Dates aligned
2015–2016	G + NDVI_vg	0.1011	0.781	Average
2015–2016	G + NDVI_vg	0.0868	0.7765	Dates aligned
2016–2017	G + NDVI_vg	0.1271	0.6354	Average
2016–2017	G + NDVI_vg	0.1602	0.6316	Dates aligned
2015–2016	G + NDVI_vg_gf	0.0989	0.7811	Average
2015–2016	G + NDVI_vg_gf	0.0867	0.7756	Dates aligned
2016–2017	G + NDVI_vg_gf	0.1462	0.6101	Average
2016–2017	G + NDVI_vg_gf	0.1707	0.6092	Dates aligned
**SVR-YLD**				
2015–2016	G	0.15	0.5577	—
2016–2017	G	0.1081	0.5304	—
2015–2016	G + NDVI_gf	0.1812	0.5638	Average
2015–2016	G + NDVI_gf	0.1464	0.5081	Dates aligned
2016–2017	G + NDVI_gf	0.1067	0.5293	Average
2016–2017	G + NDVI_gf	0.1248	0.5146	Dates aligned
2015–2016	G + NDVI_vg	0.1633	0.5563	Average
2015–2016	G + NDVI_vg	0.1602	0.4775	Dates aligned
2016–2017	G + NDVI_vg	0.0969	0.5442	Average
2016–2017	G + NDVI_vg	0.2224	0.5003	Dates aligned
2015–2016	G + NDVI_vg_gf	0.1821	0.564	Average
2015–2016	G + NDVI_vg_gf	0.1617	0.4597	Dates aligned
2016–2017	G + NDVI_vg_gf	0.1003	0.5414	Average
2016–2017	G + NDVI_vg_gf	0.2395	0.4829	Dates aligned

Results of predicting 1 entire year using the other (leave one environment out, LOO) for 2 traits TGW (thousand grains weight) and YLD (grain yield) for 2 metrics, Normalized Root Mean Squares Error Prediction (NRMSEP) and correlation (Cor Mean) between observed and predicted values for models including and genomic (G) and NDVI. Two types of NDVIs were included for NDVI vegetative (vg) and NDVI grain filling (gf), one aligning the dates (dates aligned) and the other one averaging (average) the dates. Support vector regression (SVR) by using linear kernel and default values for the rest of the parameters as done with the e1071 R package (ϵ=0.1 in the insensitive loss-function, tolerance 0.01, and cost of constraint violations equal to 1).

In terms of the GBLUP models, a contrary behavior was observed with the GBM model because in both traits, a slightly better accuracy was obtained when predicting 2015–2016 using 2016–2017 as training data when compared to the accuracy obtained by predicting 2016–2017 with 2015–2016 as training data. However, in all cases, GBM yielded lower accuracy with respect to DL models and to GBLUP when predicting 2016–2017. Gradient boosting machine also provided lower accuracy than SVR, and SVR gave slightly better accuracy than DL when predicting trait TGW in year 2015–2016 but not when predicting the same trait in year 2016–2017, resulting in DL being superior in accuracy. When predicting the second trait (YLD) in year 2015–2016, DL was superior in accuracy to SVR. The same was true when predicting the same trait in year 2016–2017 with the predictor excluding NDVIs covariables (G), the predictor average of both kind of NDVIs measurements (G + NDVI_vg_gf) and predictor including aligned NDVIs at the vegetative stage (G + NDVI_vg), or including aligned NDVIs at the grain filling stage (G + NDVI_gf).

When fitting the models G (Type of NDVI_, no NDVI used), G + NDVI_gf, G + NDVI_vg, and G + NDVI_gf_vg, the results of the metric NRMSEP for the cases of NDVI average and NDVI data aligned. Figure 2 shows the results of predicting one year using the other as a training set for both traits TGW and YLD. Most of the results show that when using NDVI for prediction with an entire year using the other year as training, the DL method had a higher GP accuracy (lower NRMSEP) than those obtained from fitting the GBLUP model. Except for when predicting genomic (G) in GBM and SVR, DL overcame the fitted GLM for trait TGW in prediction year 2016–2017 and in same year when predicting TGW with NDVI data aligned and when predicting YLD with average DNVI data, where the better accuracy was obtained with GBLUP and SVR, respectively. When predicting the trait YLD in year 2016–2017 with NDVI average, all fitted SVR models, followed by GBLUP models, had better accuracy than DL. Another exception was also found in the same context (predicting trait YLD in year 2016–2017) but with NDVI data aligned, where GBLUP models outperformed all other models; this was followed by DL model in two out three predictors.

Similarly, Fig. 3 displays the results of TGW and YLD from the metric Cor Mean when fitting the models G (Type of NDVI__, no NDVI used), G + NDVI_gf, G + NDVI_vg, and G + NDVI_gf_vg for the cases of NDVI average and NDVI data aligned and predicting one cycle using the other as training set for both traits TGW and YLD. Prediction trait TGW in year 2015–2016 with G and G + NDVI_vg for NDVI_average had higher GP accuracy for DL than GBLUP, GBM, and SVR, while for the same trait in same year but with G + NDVI_gf (average), G + NDVI_vg (dates aligned), and G + NDVI_vg_gf (average and dates aligned), SVR had higher GP accuracy than all other models (DL, GBLUP, and GBM) followed by DL. For G + NDVI_gf (dates aligned) DL and SVR had similar accuracy. Now, when predicting trait TGW in year 2016–2017, GBLUP had higher GP accuracy followed by DL, except in G + NDVI_vg_gf where DL was superior. Here, the worst performance was obtained with GBM.

Continuing with results displayed in Fig. 3, when predicting YLD in year 2015–2016 DL showed higher accuracy with G, G + NDVI_gf (dates aligned), G + NDVI_vg (dates aligned), and G + NDVI_vg_gf (average), followed by SVR, while the rest of SVR cases had a higher GP accuracy followed by DL. When predicting YLD in year 2016–2017, GBLUP had the higher accuracy followed by DL, except with G + NDVI_vg_gf (dates aligned) where DL was superior. Here again the worst behavior was obtained with GBM.

Another set of results were obtained by fitting 8 different models including year + G, and year + G + year × G combined with NDVI_vg, NDVI_gf, and NDVI_vg_gf for predicting TGW and YLD considering year + G and year + G + year × G without including NDVI and including the various types of NDVI combined as average or as dates aligned. The GP accuracy results are shown for 5FCV for metrics NRMSEP (Fig. 4) and Cor Mean (Fig. 5) for models fitted under GBLUP and DL for both traits, TGW and YLD.

Results displayed in Fig. 4 show similar GP accuracy based on NRMSEP for the 8 models with no increase in GP accuracy when adding image to the genomic (G) prediction for TGW and YLD. Another result based on 5FCV is that DL, GBM, and SVR methods slightly decreased in GP accuracy compared with the models fitted by the GBLUP for the various G and the types of NDVI combinations considered for both traits, being more notable for GBM in trait TGW and for SVR in trait YLD. Furthermore, Fig. 5 shows similar GP accuracy based on Cor Mean for the 8 models with a slight increase in GP accuracy when adding image to the genomic prediction. Also, the 5FCV based on Cor Mean showed that DL, GBM, and SVR methods slightly decreased in GP accuracy compared with the models fitted by the GBLUP for the various G and the types of NDVI combinations considered. These results also occur for TGW and YLD.

It is interesting to observe how the mean values of the optimal hyperparameters found for each DL for the 7 models for the 5FCV for both traits TGW and YLD changed for the different models (Table 9). The average values of each hyperparameter for all models were very similar, except for a few cases. For example, for trait TGW, the average weight decay of the model with predictor year + G is approximately 81 and 1,411 times the value of this hyperparameter in the rest of the 6 models (rows 3–8 Table 9), while the model with predictor year + G + NDVIs_gf and NDVIs with dates aligned, have an average value of patience hyperparameter (Pat) at least 10 units larger than the rest of the models (rows 2 and 4–8 from Table 9).

**Table 9.** DS1.

Trait	Type of NDVIs	Predictor	$N_{l}^{\left(1\right)}$	$N_{l}^{\left(2\right)}$	$N_{l}^{\left(3\right)}$	$n_{\mathrm{HL}}^{\left(1\right)}$	$n_{\mathrm{HL}}^{\left(2\right)}$	$n_{\mathrm{HL}}^{\left(3\right)}$	λ	DO	lr	wd	Pat
TGW	—	Year + G	14.6	425.6		2	2.2		0.0070	0.1545	0.0091	0.2249	27.4
TGW	Average	Year + G + NDVI_gf	13.6	654.4	43.2	2.2	2.4	1.4	0.0029	0.2204	0.0008	0.0011	38.4
TGW	Average	Year + G + NDVI_vg	11.8	403.4	53.4	2.4	1.2	3.4	0.0037	0.3175	0.0044	0.0002	27.6
TGW	Average	Year + G + NDVI_vg_gf	13.2	466.8	49.8	2.2	2.2	2.6	0.0057	0.3299	0.0007	0.0003	31.2
TGW	Dates aligned	Year + G + NDVI_gf	10.2	625.6	39.4	1.6	2.6	2.8	0.0043	0.1791	0.0023	0.0028	38.6
TGW	Dates aligned	Year + G + NDVI_vg	11.2	625.8	43	2	1.4	2.4	0.0066	0.2521	0.0018	0.0002	37.8
TGW	Dates aligned	Year + G + NDVI_vg_gf	12.8	521.4	47.8	2.2	2.8	2.4	0.0063	0.2482	0.0031	0.0009	37.2
YLD	—	Year + G	11.6	432.2		1.8	4.2		0.0011	0.1387	0.0064	0.2460	30
YLD	Average	Year + G + NDVI_gf	11.6	125.8	55.8	1.4	2.6	3.8	0.0054	0.2687	0.0029	0.0059	42.4
YLD	Average	Year + G + NDVI_vg	9.2	551.4	40.6	1.4	2.4	4.2	0.0054	0.2456	0.0014	0.0005	39.8
YLD	Average	Year + G + NDVI_vg_gf	13	244.2	51.4	1.8	2	3	0.0040	0.1565	0.0007	0.0020	38
YLD	Dates aligned	Year + G + NDVI_gf	11.6	241.6	52.6	2	2.2	3.4	0.0064	0.3028	0.0021	0.0038	31.6
YLD	Dates aligned	Year + G + NDVI_vg	13	383.2	43.2	2	1.8	3	0.0061	0.2198	0.0024	0.0078	32.6
YLD	Dates aligned	Year + G + NDVI_vg_gf	9.8	468.2	49.6	1.8	3.4	2.8	0.0059	0.0884	0.0039	0.0044	38.2

Average across partitions of the optimal hyperparameters values found for each multimodal DL models in the 5-fold cross-validation (5FCV) evaluation performance strategy. Empty cell means do not apply.

For trait YLD, a similar but a slightly more ambiguous pattern was observed in the hyperparameters shared by all models (rows 9–15 Table 9). For example, the average neuron values of models with predictors year + G + NDVIs_gf and year + G + NDVIs_vg_gf, both with dates aligned, differed more than the rest of models, with at least 226 units. We also observed a notable difference across models in the average number of hidden layers for input 2 (G), ranging from 1.2 to 4.2, and the larger average of hidden layers was obtained in the first model (row 9), which is near of the upper bound (see Table 1) of the specified domain space for such a hyperparameter. In this same model (row 9) the average weight decay value was observed to differ greatly with respect to the other models, between 31 and 520 times greater.

Similarly, for the LOO (one year predicting all the lines in another year) genomic prediction method, the values of the optimal hyperparameters found are shown in Table 10 for each trait, for each model, and for each predicted year, where a greater variation can be appreciated with respect to the 5FCV case.

**Table 10.** DS1.

Trait	Predictor	Type of NDVIs	Year	$N_{l}^{\left(1\right)}$	$N_{l}^{\left(2\right)}$	$n_{\mathrm{HL}}^{\left(1\right)}$	$n_{\mathrm{HL}}^{\left(2\right)}$	λ	DO	lr	wd	Pat
TGW	G	—	2016–2017		189		2	0.0008	0.3943	0.0028	0.0001	33
TGW	G	—	2015–2016		188		1	0.0100	0.0001	0.0100	0.0000	32
TGW	G + NDVI_gf	Average	2016–2017	16	225	1	1	0.0100	0.0001	0.0100	0.4000	43
TGW	G + NDVI_gf	Average	2015–2016	6	905	1	4	0.0090	0.2574	0.0017	0.0193	59
TGW	G + NDVI_vg	Average	2016–2017	14	819	3	4	0.0049	0.1831	0.0005	0.0002	36
TGW	G + NDVI_vg	Average	2015–2016	15	701	2	1	0.0068	0.1698	0.0016	0.0118	25
TGW	G + NDVI_vg_gf	Average	2016–2017	16	570	4	1	0.0100	0.5000	0.0100	0.4000	64
TGW	G + NDVI_vg_gf	Average	2015–2016	16	914	3	1	0.0028	0.5000	0.0100	0.0001	43
TGW	G + NDVI_gf	Dates aligned	2016–2017	15	566	1	5	0.0008	0.2735	0.0033	0.0001	24
TGW	G + NDVI_gf	Dates aligned	2015–2016	14	101	3	2	0.0042	0.0196	0.0075	0.0054	11
TGW	G + NDVI_vg	Dates aligned	2016–2017	16	356	1	1	0.0100	0.0001	0.0100	0.4000	22
TGW	G + NDVI_vg	Dates aligned	2015–2016	16	338	1	1	0.0000	0.5000	0.0100	0.4000	64
TGW	G + NDVI_vg_gf	Dates aligned	2016–2017	16	437	1	6	0.0100	0.0001	0.0100	0.4000	26
TGW	G + NDVI_vg_gf	Dates aligned	2015–2016	9	926	3	4	0.0037	0.1549	0.0045	0.0146	35
YLD	G	—	2016–2017		970		3	0.0060	0.3977	0.0000	0.0001	11
YLD	G	—	2015–2016		743		1	0.0100	0.5000	0.0100	0.0000	16
YLD	G + NDVI_gf	Average	2016–2017	16	944	4	1	0.0000	0.0001	0.0100	0.4000	64
YLD	G + NDVI_gf	Average	2015–2016	16	916	1	6	0.0000	0.0001	0.0100	0.4000	64
YLD	G + NDVI_vg	Average	2016–2017	16	937	4	1	0.0100	0.5000	0.0100	0.4000	46
YLD	G + NDVI_vg	Average	2015–2016	14	682	3	2	0.0050	0.0456	0.0079	0.1790	22
YLD	G + NDVI_vg_gf	Average	2016–2017	9	505	2	4	0.0020	0.3647	0.0003	0.0001	39
YLD	G + NDVI_vg_gf	Average	2015–2016	12	797	1	1	0.0035	0.0001	0.0003	0.0008	23
YLD	G + NDVI_gf	Dates aligned	2016–2017	12	128	3	3	0.0049	0.2760	0.0045	0.1088	53
YLD	G + NDVI_gf	Dates aligned	2015–2016	15	269	3	4	0.0068	0.2249	0.0012	0.0010	59
YLD	G + NDVI_vg	Dates aligned	2016–2017	13	908	3	5	0.0018	0.1830	0.0002	0.0002	8
YLD	G + NDVI_vg	Dates aligned	2015–2016	11	887	1	3	0.0025	0.0770	0.0008	0.1558	48
YLD	G + NDVI_vg_gf	Dates aligned	2016–2017	15	411	1	3	0.0078	0.0307	0.0037	0.0007	62
YLD	G + NDVI_vg_gf	Dates aligned	2015–2016	16	928	1	1	0.0100	0.5000	0.0100	0.4000	64

Optimal hyperparameter values found for each DL model when predicting 1 entire year (year) using all the lines from the other year (leave one environment out, LOO). An empty cell means it does not apply.

### Results DS2

The results where each row panel corresponds to each trait and column panel for the predicted environment are shown in Fig. 6 (NRMSEP) and Fig. 7 (Cor). When predicting the irrigated environment with the drought environment, DL had higher accuracy (less NRMSEP) than the GBLUP model in all analyzed traits and all years. The most considerable differences were obtained in traits YLD and BM (Fig. 6). When predicting drought environment with information on the irrigated environment, the DL model and GBLUP model had similar accuracy except in the first year (2015–2016) where a more significant difference was obtained.

A more ambiguous pattern is found regarding the Pearson's correlation metric (Cor, Fig. 7). For trait BM, when predicting the drought environment with the irrigated environment in 2 of the 3 years, DL had higher accuracy, while when predicting the irrigated environment, in 2 of the 3 years GBLUP model had higher accuracy. However, in 1 of these years, the difference was marginal. For trait HI, the GBLUP model was more accurate than DL when predicting drought environment. When predicting this same trait in the irrigated environment, DL resulted better in 1 of the 2 years (2015–2016 and 2016–2017). For trait PMG, GBLUP resulted better than DL in all cases but with a marginal difference in both years when predicting drought environment. For the last trait (YLD), the DL model was less accurate than the GBLUP model when predicting the drought environment, but in 2 of the 3 years, the difference was less. When predicting this same trait in the irrigated environment, in 2 of the 3 years, DL was better.

## Discussion

### Bases of the novelty of the proposed multimodal DL

The novelty of the DL developed in this study includes 3 modalities corresponding to the three types of input data: genomic, year, and image. The superiority, in some scenarios, of the DL methods in part can be attributed to the novel architecture used in the deep learning models implemented. The novelty of the architecture is due to three components.

The first component is the architecture of multimodal deep learning models (MMDL; see Fig. 1) with three-types of inputs corresponding to the effects of environments ($X_{\mathrm{year}})$, the genomic information ($Z_{L}^{*}=Z_{L}L^{T})$, and the effect of NDVIs ($X_{\mathrm{NDVIs}})$. For each modality (type of input), a separate DL model is trained in the initial phase, and in a second stage, the outputs of all 3 DL models are concatenated, forming another MP model with few layers. Finally, from this model, predictions are obtained. The training process of this multimodal DL model is done simultaneously; however, it is more challenging since a careful tuning process needs to be implemented to guarantee reasonable predictions. Nevertheless, multimodal learning helps researchers understand and analyze when various sensors are engaged in the processing of information. For this reason, multimodal DL have been successfully applied to deal with multiple types of modalities, i.e. image, video, text, audio, body gestures, facial expressions, and physiological signals. Because multimodal DL models can combine multiple heterogenous sources of information, prediction accuracy is improved (Ramachandram and Taylor 2017).

The second novelty of the multimodal DL used in this study is that it belongs to residual networks (ResNet) (He *et al*. 2016), which reduce (or avoid) the vanishing gradient problem, where the gradient is back-propagated to earlier layers and becomes infinitively small. As a result, as the network goes deeper, its performance gets saturated or even starts degrading rapidly, making it difficult to train. Therefore, it is not enough to simply stack layers together. ResNet architectures allow us to successfully train deep neural networks up to hundreds or even thousands of layers since it introduces the so-called “identity shortcut connection” that skips one or more layers; therefore, this technique reduces or avoids the vanishing gradient problem. Under ResNet, stacking layers should not degrade the network performance, since “identity shortcut connection” simply stacks identity mappings (layers that are not of any use; He *et al.* 2016). For this reason, ResNet architectures have a powerful representational ability employed in many regression and classification problems.

The third novelty of this research refers to the tuning process of the hyperparameters under Bayesian optimization using the function (library bayes_opt), which does not perform an exhaustive search of the space like grid search and random search that evaluate all (or some) hyperparameter configurations resulting from the cartesian product of the discretized search space of each hyperparameter. Bayesian optimization (Mockus 2012) is more efficient since it builds a probabilistic model for a given function and analyzes this model to decide where to next evaluate the given function. The two main components of Bayesian optimization are (1) a prior function that captures the main patterns of the unknown objective function and a probabilistic model that describes the data generation mechanism and (2) an acquisition function (loss function) that describes how optimal a sequence of queries is.

Our results show that even with small- to medium-sized data sets, the deep learning methodology is very competitive. Nevertheless, using more sophisticated architectures takes advantage of the data and deep learning methodology. Validation on additional data must be done to better understand the behavior of the multimodal DL model. This includes larger data sets with denser NDVI data to combine different types of NN distinct from MP as was used here in each modality. Other late fusion strategies (Ramachandram *et al*. 2017; Baltrušaitis *et al*. 2018) also need to be explored, such as weighing the individual outputs based on its uncertainty as proposed by Wang *et al*. (2021), or including more layers after the individual outputs of each sub-model are combined (Huang *et al*. 2020) with the potential advantage of learning interactions between features of different modalities (Stahlschmidt *et al*. 2022).

### Comparing multimodal DL with other results

Interestingly, Danilevicz *et al*. (2021) employed multimodal DL using 2 tabular inputs (modules), 1 module has information from the parents, the seed stock, fertilizer, and planting date (using a sigmoid layer), and the other is a spectral module (employing a linear layer). The weights from each of these 2 modules were concatenated, and their weights were combined in the fusion module that produces the outputs of the multimodal yield prediction. The multimodal DL proposed by Danilevicz *et al*. (2021) functions as a support tool for early identification of good maize lines. However, the authors noted that since the model was trained in a single location data (and thus no GE was incorporated into the multimodal DL), further training with new data from other environments would be required to improve the prediction accuracy. The recent study conducted by Kick *et al*. (2023) integrates genomics, environments, and management into the multimodal DL and examined the effect of various types of interactions. Results show that although the DL and GBLUP provided similar prediction accuracy, the GBLUP has the lowest average error, whereas the multimodal DL performed more consistently with similar average errors.

Our study shows that the prediction of cultivars in new environments and future years is one of the key issues in genomic-enabled prediction. The results from the DL proposed in this study for the prediction accuracy of new years are similar or higher than those obtained from the GBLUP when using genomic and high-throughput phenotype (image) for traits’ grain yield and thousand kernel weight from DS1 and grain yield under irrigation for DS2. Support vector regression also performs very well when predicting grain yield in future years.

### Conclusions

In this study, we present outcomes from some genomic prediction models (GBLUP, GBM, and SVR) and compared them with the novel DL method using a wheat data set comprised of genomic and high-throughput phenotype (images) for 2 traits measured in 2 years on multiple wheat lines (DS1). Results show that the novel DL approach presented in this study was accurate for predicting 1 year based on the other one (LOO) and outperformed the other models for both YLD and TGW traits, NDVI average and NDVI dates aligned, and at both metrics used to assess the GP accuracy (NRMSPE and Cor Mean). However, GP accuracy results obtained for the 5FCV indicated that the GBLUP model in DS1 was slightly superior to the DL, when genomic and various types of NDVI measurements were included. The genomic prediction of 4 traits (BM, HI, PMG, and YLD) for drought and irrigation in 3 years (DS2) show slight increases in GP accuracy of DL over GBLUP for traits HI and YLD under irrigated conditions for some years but not for the 3 years under drought conditions. Results from this study show that the multimodal DL method has a robust degree of generalization with other very data-specific DL previously reported. It is also important to stress that the GBLUP R package is a very vigorous parametric statistical software for GP accuracy. The DL method used in this study is novel and presents a good degree of generalization and important accuracy for predicting new years. One reason is that instead of concatenating all feature types and using them to feed the created network, the outputs are combined to create the output value for each type of information and individual neural network.

## Data Availability

The phenotypic and genomic data corresponding to the two data sets used in this study (DS1 and DS2) can be downloaded from the following link https://hdl.handle.net/11529/10548885.

## Appendix

### Adjusting the phenotypic DS1

The Best Linear Unbiased Estimates (BLUE) of each wheat lines for trait, GY, and TGW and for each of the wavelengths were obtained at each of the time-points using the following model:

$$y_{ijkl}=\mu +g_{i}+t_{j}+r_{k(j)}+b_{l(k,j)}+\epsilon_{ijkl},$$

where μ is the overall mean; $y_{ijkl}$ is the response variable (GY, TGW, and wavelength) for the *i* th wheat line, at the *k* th replicate, of the *l* th block at the *j* th trial; $g_{i}$ is the effect of the *i*th wheat line; $t_{j}$ is the effect of the trial; $r_{k(j)}$ is the random effect of replicate *k* th nested within trial jth with normal distribution $N(0,\sigma_{r}^{2})$ where $\sigma_{r}^{2}$ is the variance of the replicate; $b_{l(k,j)}$ is the random effect of the incomplete block lth nested within replicate *k*th and trial jth with normal distribution $N(0,\sigma_{b}^{2})$, where $\sigma_{b}^{2}$ is the variance of the incomplete block; and $\epsilon_{ijkl}$ is the residual effect with normal distribution $N(0,\sigma_{e}^{2})$ where $\sigma_{e}^{2}$ is the error variance. After making these pre-adjustments in each year, we obtained BLUE for each of the 350 wheat lines for traits GY and TGW and for each of the 250 bands for each time-points under study.

### Adjusting the phenotypic DS2

The design effect of each environment-year combination considered and the BLUE of each wheat lines were estimated with the following model:

$$y_{ijkl}=\mu +g_{i}+t_{j}+r_{k(j)}+b_{l(k,j)}+\epsilon_{ijkl},$$

where μ is the overall mean; $y_{ijkl}$ is the response variable for the *i* th wheat line, at the *k* th replicate, and *l* th block of the *j* th trial; $g_{i}$ is the effect of the *i*th wheat line; $t_{j}$ is the effect of trial; $r_{k(j)}$ is the random effect of replicate *k* th nested within trial jth with normal distribution $N(0,\sigma_{r}^{2})$, where $\sigma_{r}^{2}\mathrm{is}\;\mathrm{the}\;\mathrm{variance}\;\mathrm{of}\;\mathrm{the}\;\mathrm{replicate}$$b_{l(k,j)}$ is the random effect of the incomplete block lth nested within replicate *k*th and trial jth with normal distribution $N(0,\sigma_{b}^{2})$, where $\sigma_{b}^{2}$ is the variance of the incomplete block; and $\epsilon_{ijkl}$ is the residual effect with normal distribution $N(0,\sigma_{e}^{2})\;{\mathrm{where}\;\sigma }_{e}^{2}\;\mathrm{is}\;\mathrm{the}\;\mathrm{error}\;\mathrm{variance}$.
