# Correction to: Multimodal deep learning methods enhance genomic prediction of wheat breeding

**DOI:** 10.1093/g3journal/jkad081

**Published:** 2023-04-13

This is a correction to: Abelardo Montesinos-López, Carolina Rivera, Francisco Pinto, Francisco Piñera, David Gonzalez, Mathew Reynolds, Paulino Pérez-Rodríguez, Huihui Li, Osval A Montesinos-López, Jose Crossa, Multimodal deep learning methods enhance genomic prediction of wheat breeding, *G3 Genes*|*Genomes*|*Genetics*, Volume 13, Issue 5, May 2023, jkad045, https://doi.org/10.1093/g3journal/jkad045

In the originally published version of this manuscript, author Huihui Li's name was given as H.Li.

This error has been corrected.

